# IRF1 Inhibits Therapy-Induced Senescence of Glioblastoma Cells Through OAS2

**DOI:** 10.3390/cells15131149

**Published:** 2026-06-24

**Authors:** Genli Ma, Weiwei Huang, Hangcong Yan, Bin Shen, Shanli Zhu, Xingxing Xu

**Affiliations:** School of Basic Medical Sciences, Wenzhou Medical University, Wenzhou 325035, China; maggie0907@wmu.edu.cn (G.M.); huangweiwei@wmu.edu.cn (W.H.); yanhangcong@wmu.edu.cn (H.Y.); shenbin@wmu.edu.cn (B.S.)

**Keywords:** glioblastoma, senescence, IRF1, OAS2

## Abstract

**Highlights:**

**What are the main findings?**

- Downregulation of IRF1 promotes senescence of glioblastoma (GBM) cells (increased SA-β-gal activity, decreased Lamin B1 expression, reduced cell proliferation, and enhanced SASP).
- IRF1 positively regulates OAS2, and both are downregulated in senescent GBM cells; knockdown of OAS2 mimics IRF1 knockdown, showing that IRF1 acts through OAS2 to inhibit senescence.

**What are the implications of the main findings?**

- The identification of the IRF1–OAS2 axis as a novel regulator of therapy-induced senescence (TIS) provides mechanistic insight into GBM relapse and suggests that pharmacological modulation of this pathway could enhance TMZ efficacy and reduce recurrence of GBM.

**Abstract:**

Glioblastoma (GBM), a highly aggressive brain tumor, is characterized by poor treatment outcomes and a strong tendency to recur after therapy. Therapy-induced senescence (TIS) of GBM cells has emerged as a key driver of GBM progression and relapse. Temozolomide (TMZ), which serves as the standard chemotherapeutic agent for GBM, is known to induce senescence; however, the molecular mechanisms underlying this process remain largely unknown. In this work, we found that interferon regulatory factor-1 (IRF1) was downregulated in TMZ-induced senescent GBM cells. Functionally, knockdown of IRF1 increased the activity of senescence-associated β-galactosidase (SA-β-gal), reduced protein expression of Lamin B1, inhibited cell division, and enhanced senescence-associated secretory phenotype (SASP) of GBM cells, indicating that downregulation of IRF1 promotes senescence of GBM cells. Conversely, overexpression of IRF1 partially reversed TMZ-induced senescence. Further exploration revealed that downregulation of IRF1 reduced the expression of 2′,5′-oligoadenylate synthetase 2 (OAS2), and overexpression of IRF1 increased the expression of OAS2. OAS2 was also downregulated in TMZ-induced senescent GBM cells, and knockdown of OAS2 induced senescence of GBM cells as well. Taken together, our study reveals that IRF1 inhibits TMZ-induced senescence of GBM cells through OAS2, highlighting a novel regulatory axis that may offer potential therapeutic targets for improving GBM treatment.

## 1. Introduction

Glioblastoma (GBM) accounts for approximately 48.3% of central nervous system (CNS) malignant tumors [1], serving as the predominant contributor to CNS-related cancer mortality, with a five-year survival rate of only about 5% [2]. Currently, standard therapy includes surgical resection followed by radiotherapy and chemotherapy, with temozolomide (TMZ) as the first-line chemotherapeutic drug [3,4].

Recent studies have demonstrated that senescence of GBM cells occurs following chemotherapy and radiotherapy—a phenomenon termed therapy-induced senescence (TIS), which in turn contributes to the progression and recurrence of GBM [5,6,7,8,9,10]. Cellular senescence represents a complicated process marked primarily by inhibition of cell proliferation and appearance of senescence-associated secretory phenotype (SASP); the latter refers to active secretion of pro-inflammatory cytokines and chemokines, along with growth factors and matrix metalloproteinases (MMPs) in response to various stressors [11,12,13].

Studies have shown that some immune signaling molecules are involved in senescence of GBM cells, such as IFI16, STAT1/3 [7,14,15,16]. As a critical transcription factor in the interferon immune pathway, interferon regulatory factor-1 (IRF1) controls the expression of interferon-stimulated genes, and plays major roles in cell proliferation, apoptosis, stress response, DNA damage response and immune response [17,18,19,20,21].

In recent years, the function of IRF1 in the process of cellular senescence has been investigated by several studies. After irradiation, the level of IRF1 is increased in human GBM cells and human structural cells [21,22]. Irradiation also induces nuclear translocation and activation of IRF1 [21]. Overexpression of IRF1 enhances radiation-induced senescence, whereas knockdown of IRF1 rescues radiation-induced senescence in skin cells [21]. Additionally, the expression of IRF1 is elevated in human melanoma cells following interferon treatment, and interferon treatment triggers cellular senescence of endothelial cells [23]. These studies suggest that IRF1 promotes cellular senescence. However, one study reported that knockdown of IRF1 increased senescence of chondrocytes [24]. Despite the fact that the function of IRF1 in modulating cellular senescence has received considerable attention, the results remain controversial, and its specific role in the cellular senescence of GBM cells is still unclear.

As a transcription factor, IRF1 binds to interferon-stimulated response elements to regulate multiple downstream genes, including *oas2* [25,26,27]. 2′,5′-oligoadenylate synthetase 2 (OAS2) is involved in antiviral responses and cell growth regulation [28,29]. Recent evidence suggests that OAS2 can modulate cellular senescence [30,31], but its role in GBM cell senescence has not been explored.

In this work, we investigated the functional role of IRF1 in TMZ-induced senescence of GBM cells and tested the hypothesis that IRF1 acts through OAS2 to suppress senescence. Our findings reveal a previously unrecognized IRF1-OAS2 axis that inhibits TIS, offering potential targets to improve GBM treatment.

## 2. Materials and Methods

The small interfering RNAs (siRNAs): The siRNAs targeting IRF1 and OAS2 were synthesized by Sangon Biotech (Shanghai, China). IRF1-siRNA sequence is 5′-UAGUGUACACCUCUGAUCA-3′ [32]; the control non-silencing sequence is 5′-UUCUCCGAACGUGUCACGUTT-3′. OAS2-siRNA sequence is 5′-AAGCAGGGAGAGGAUAACC-3′; the control non-silencing sequence is 5′-UUCUCCGAACGUGUCACGU-3′ [29]. The stock concentration of siRNAs was 100 μM.

Lentivirus: The control and IRF1-overexpressing lentiviruses were synthesized by Obio Technology (Shanghai, China). Human IRF1 (Gene ID: 3659) was cloned into the pSLenti-EF1-EGFP-F2A-Puro-CMV-Myc-WPRE vector, and the resulting pSLenti-EF1-EGFP-F2A-Puro-CMV-IRF1-Myc-WPRE vector was packaged into lentivirus to generate IRF1--overexpressing lentivirus. Similarly, the control lentivirus was generated using this pSLenti-EF1-EGFP-F2A-Puro-CMV-Myc-WPRE vector.

Cell culture and transfection: GBM cell line DBTRG-05MG was purchased from Nanjing Kebai Biotechnology (Nanjing, China), and U251 cells were kindly provided by Professor Feng Tan (Wenzhou Medical University, Wenzhou, China). They were grown in DMEM (C11995500BT, Thermo Fisher Scientific, Waltham, MA, USA) containing 10% FBS (A5256701, Thermo Fisher Scientific) and 1% penicillin/streptomycin (C100C5, Thermo Fisher Scientific). Cells were maintained in a humidified incubator with 5% CO_2_ at 37 °C.

Cells were transfected with the corresponding siRNAs at approximately 200 nM using Lipofectamine 2000 (11668-019, Thermo Fisher Scientific), following the manufacturer’s protocol. Transfected cells were harvested for subsequent assays.

TIS model of GBM cells: Senescence of DBTRG and U251 cells was induced by TMZ (HY-17364, MedChemExpress, Monmouth Junction, NJ, USA), and TMZ at concentrations of 25–100 μM was applied for 4–8 days [9]. A 100 mM stock solution of TMZ was obtained by dissolving the compound in DMSO. The final concentration of DMSO was less than 0.1%. When cells were treated with TMZ alone (without viral infection), confluency was maintained at 30–40%; when TMZ treatment was combined with viral infection, confluency was kept at 40–50%. Senescent GBM cells were identified by senescence-associated β-galactosidase (SA-β-gal) staining.

SA-β-gal staining: Staining was performed according to the detailed instructions of the SA-β-gal staining kit (G1580, Solarbio, Beijing, China) [33]. The stained cells were mounted using an anti-fading mounting medium (S2110, Solarbio). Observation was performed under a light microscope (Olympus, Tokyo, Japan). Blue signals appeared in SA-β-gal-positive cells. Senescence levels of GBM cells were evaluated based on the proportion of SA-β-gal^+^ cells, i.e., SA-β-gal^+^ cells (%), which was defined as the ratio of blue-stained cells to total cells (determined by DAPI staining).

RNA extraction and quantitative real-time PCR (qRT-PCR): Total RNA was extracted using TRIzol reagent (15596026, Thermo Fisher Scientific), and the first-strand cDNA was synthesized from 1 to 2 µg of RNA using a reverse transcription kit (638313, TaKaRa, Kusatsu, Japan). qPCR was performed on the CFX96 detection system (Bio-Rad, Hercules, CA, USA) using the TB Green qPCR kit (CN830A, TaKaRa). Each reaction was run in triplicate, and relative expression levels were calculated using the 2^−ΔΔCt^ method after normalization to the *gapdh* reference gene. The primers used in this study were as follows: IRF1-F, 5′-GAGGAGGTGAAAGACCAGAGCA-3′; IRF1-R, 5′-TAGCATCTCGGCTGGACTTCGA-3′, GAPDH-F, 5′-ATCAAGAAGGTGGTGAAGCA-3′; GAPDH-R, 5′-GTCGCTGTTGAAGTCAGAGGA-3′ [34]. The primers were synthesized by Sangon Biotech (Shanghai, China).

Western blot: Lysis of the cells was carried out on ice using RIPA buffer (WB3100, NCM, Suzhou, China) containing PMSF (P0100, Solarbio), protease inhibitor cocktail (P001, NCM), Na_3_VO_4_ and NaF. The lysates were then transferred into tubes and placed on a mixing rotator for about 45 min at 4 °C. After centrifugation (12,000 *g* for 20–30 min), the supernatant was combined with 5 × loading buffer (P1040, Solarbio) and denatured at 100 °C for 8–10 min. Protein separation was performed by 10% SDS-PAGE, and then the proteins were transferred onto an activated PVDF membrane (IPVH00010, Millipore, Burlington, MA, USA). Thereafter, the membrane was incubated for 2 h in blocking buffer, followed by three washes with 1 × TBST (15 min each). The blocking buffer used was 1 × TBST containing 5% skim milk (232100, BD, Franklin Lakes, NJ, USA). Next, incubation with primary antibodies was conducted on a shaker overnight at 4 °C. The following primary antibodies were used: rabbit anti-Lamin B1 (ab16048, Abcam, Cambridge, UK, 1:1000), rabbit anti-IRF1 (8478S, CST, Danvers, MA, USA, 1:1000), rabbit anti-OAS2 (19279-1-AP, Proteintech, Rosemont, IL, USA, 1:1000), and mouse anti-GAPDH (ab8245, Abcam, 1:20,000). The next day, after three washes with 1 × TBST (15 min each), incubation with secondary antibodies was performed at room temperature for 2 h. The secondary antibodies used were goat anti-mouse IgG (31430, ThermoFisher Scientific) and goat anti-rabbit IgG (31460, Thermo Fisher Scientific). Afterwards, the three-time wash step was repeated. Detection of protein bands was carried out with an ECL kit (P10300, NCM), and quantitative analysis of protein expression was performed using Image J (version 1.54, National Institutes of Health, Bethesda, MD, USA).

Immunocytochemistry: Cells were first rinsed once, followed by fixation with 4% paraformaldehyde for about 20 min. Afterwards, 1 × PBS solution was applied to wash the cells three times, each for 5 min. Next, blocking and permeabilization were carried out with 0.1% PBST (0.1% Triton X-100 in PBS) containing 5% BSA. Subsequently, incubation with primary antibodies was conducted overnight at 4 °C. The following primary antibodies were used: rabbit anti-IRF1 (8478S, CST, 1:200), rabbit anti-OAS2 (19279-1-AP, Proteintech, 1:200), mouse anti-phosphorylated histone H3 (PH3) (ab14955, Abcam, 1:250) and mouse anti-GAPDH (ab8245, Abcam, 1:300). On the following day, the cells underwent three alternating washes with PBS and 0.1% PBST, each for 5 min. Then, incubation with secondary antibodies was performed at room temperature for 2 h in the dark. The secondary antibodies used were anti-rabbit Alexa Fluor488 (A21206, Thermo Fisher Scientific, 1:1000), anti-mouse Alexa Fluor594 (715-585-150, Jackson, West Grove, PA, USA, 1:1000), and anti-mouse Alexa Fluor546 (A10036, Thermo Fisher Scientific, 1:1000). After another three washes, the stained cells were mounted using an anti-fading mounting medium (S2110, Solarbio). Images were collected via a fluorescence microscope (Olympus, Tokyo, Japan) and quantitative analysis was performed by using Image J.

Enzyme-linked immunosorbent assay (ELISA): ELISA kits for IL-6 (JL14113, JONLNBIO, Shanghai, China) and MMP-3 (JL10218, JONLNBIO) were applied to determine their concentrations in the supernatant, following the manufacturer’s instructions. Firstly, the supernatants were centrifuged at 1000× *g* for 20 min, then appropriately diluted according to the expected concentration range of the samples. After equilibration of the plate strips, aliquots (100 μL/well) of test samples or standards were added, while blank wells were supplemented with universal diluent (100 μL/well). After incubation at 37 °C for 60 min, the liquid was removed and biotinylated detection antibody working solution (100 μL/well) was added. Repeated the incubation step. Wells were then washed three times with wash buffer (300 μL/well). Afterwards, enzyme conjugate working solution (100 μL/well) was added, followed by incubation at 37 °C for 30 min. After five washes, TMB substrate solution (90 μL/well) was added, and incubation was performed at 37 °C in the dark for 15 min. Finally, the stop solution (50 μL/well) was added and the absorbance (OD value) was immediately measured at 450 nm.

Statistical analysis: GraphPad Prism 8.0 (San Diego, CA, USA) was applied for statistical analysis. Data presentation: mean ± SEM. Normality assessment of the data was carried out using the Shapiro–Wilk test. The *n* value indicates the number of biological replicates. Two-group mean comparison: paired Student’s *t*-test. Multiple-group comparison: one-way ANOVA. Statistical significance: *p* < 0.05.

## 3. Results

### 3.1. IRF1 Is Downregulated in TMZ-Induced Senescent GBM Cells

**Figure 1:**
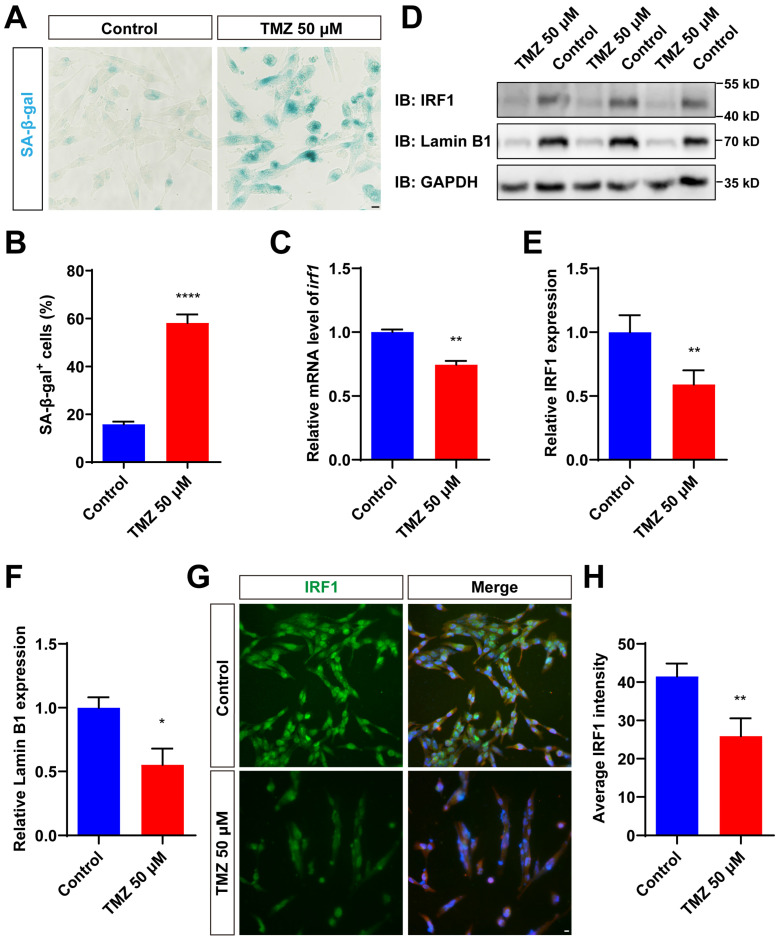
Interferon regulatory factor-1 (IRF1) is downregulated in temozolomide (TMZ)-induced senescent glioblastoma (GBM) cells. (**A**) Representative senescence-associated β-galactosidase (SA-β-gal) staining micrographs of DBTRG cells exposed to either DMSO or 50 μM TMZ over 4–8 days. (**B**) Quantitative analysis of the proportion of SA-β-gal^+^ DBTRG cells (*n* = 6). **(C)** The mRNA level of IRF1 in DBTRG cells exposed to either DMSO or 50 μM TMZ over 4–8 days, as detected by quantitative real-time PCR (qRT-PCR). (**D**) Expression of IRF1 and Lamin B1 in DBTRG cells exposed to either DMSO or 50 μM TMZ over 4–8 days, as detected by Western blot. (**E**,**F**) Quantitative analysis of Lamin B1 and IRF1 expression (*n* = 5). (**G**) Representative immunofluorescence images of IRF1 (green) and GAPDH (red) in control and TMZ-induced senescent DBTRG cells. (**H**) Quantitative analysis of average IRF1 fluorescence intensity in DBTRG cells (*n* = 5). Nuclei were labeled with DAPI (blue). Student’s *t*-test. * *p* < 0.05, ** *p* < 0.01, **** *p* < 0.0001. Scale bar, 20 μm.

To explore the potential role of IRF1 in therapy-induced senescent GBM cells, TMZ, the primary chemotherapeutic drug against GBM [35], which mainly induces senescence of GBM cells [9,36,37], was used to establish a TIS model of GBM cells. Two studies showed that treatment with low-concentration TMZ for 4–8 days predominantly induces senescence of GBM cells [37,38]. Similarly, in our study, after treatment with 25 and 50 μM TMZ for 4–8 days, a significant increase was observed in the proportion of SA-β-gal-positive DBTRG cells, with 50 μM TMZ inducing a higher percentage of senescent DBTRG cells (Figure 1A,B and Supplementary Figure S1A,B). However, when 50 μM TMZ was applied twice, the proportion of SA-β-gal-positive DBTRG cells tended to decrease, compared with DBTRG cells treated with TMZ only once (Supplementary Figure S1C,D), possibly because more GBM cells underwent apoptosis under such a condition. After 50 μM TMZ treatment, the growth of DBTRG cells gradually decreased over time (Supplementary Figure S2A), and cell proliferation was attenuated as well, as indicated by PH3, (a cell proliferation marker) staining (Supplementary Figure S2B,C). The mRNA level of IRF1 was decreased in DBTRG cells after 50 μM TMZ treatment (Figure 1C). Reduced IRF1 expression was detected in 50 μM TMZ-elicited senescent DBTRG cells and the expression of Lamin B1 (a senescent marker) was also decreased, as shown by Western blot (Figure 1D–F and Supplementary Figure S3A). Moreover, immunostaining confirmed reduced expression of IRF1 following TMZ treatment (Figure 1G–H). Collectively, these data suggest that IRF1 is downregulated in TMZ-induced senescent GBM cells.

### 3.2. Knockdown of IRF1 Induces Senescence and Promotes the Secretion of SASP Factor of GBM Cells

**Figure 2:**
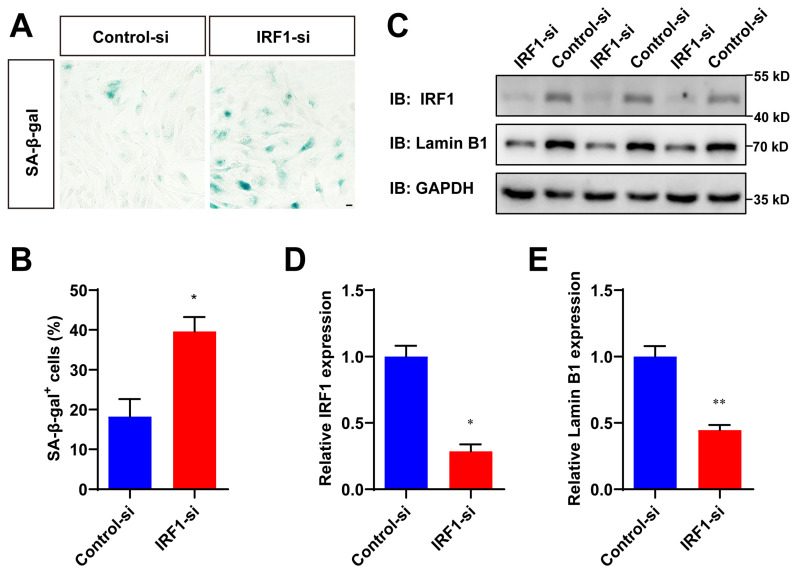
Downregulation of IRF1 promotes senescence of GBM cells. (**A**) Representative SA-β-gal staining micrographs of DBTRG cells transfected with either control- small interfering RNA (siRNA) or IRF1-siRNA for 48–96 h. (**B**) Quantitative analysis of the proportion of SA-β-gal^+^ DBTRG cells (*n* = 4). (**C**) Expression of IRF1 and Lamin B1 in DBTRG cells transfected with either control-siRNA or IRF1-siRNA for 48–96 h, as detected by Western blot. (**D**,**E**) Quantitative analysis of IRF1 and Lamin B1 expression (*n* = 3). Student’s *t*-test. * *p* < 0.05, ** *p* < 0.01. Scale bar, 20 μm.

**Figure 3:**
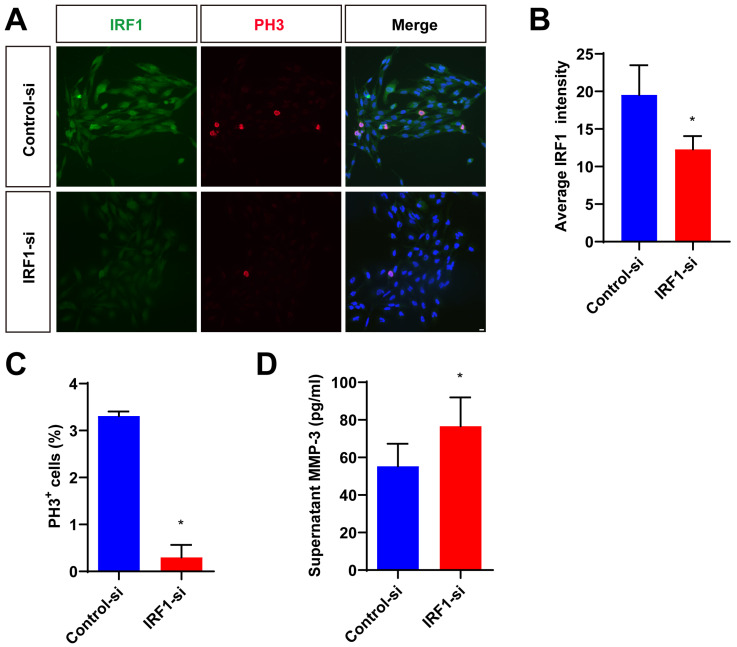
Downregulation of IRF1 inhibits cell proliferation and promotes the secretion of senescence-associated secretory phenotype (SASP) factor of GBM cells. (**A**) Representative IRF1 (green) and phosphorylated histone H3 (PH3, red) staining images of DBTRG cells transfected with either control-siRNA or IRF1-siRNA for 48–96 h. (**B**,**C**) Quantitative analysis of average fluorescence intensity of IRF1 (*n* = 6) and the proportion of PH3^+^ cells (*n* = 3). (**D**) Measurement of MMP-3 level in the supernatant of DBTRG cells transfected with either control-siRNA or IRF1-siRNA for 48–96 h, as detected by enzyme-linked immunosorbent assay (ELISA) (*n* = 6). Nuclei were stained with DAPI (blue). Student’s *t*-test. * *p* < 0.05. Scale bar, 20 μm.

To further examine the specific function of IRF1 during GBM senescence, IRF1-specific siRNA was synthesized to knock down IRF1 [32]. As shown in Figure 2A,B, a decrease in the proportion of SA-β-gal-positive DBTRG cells was detected after knockdown of IRF1, compared with control cells, and some cells were also found to be dead, indicating that IRF1 knockdown induced cellular senescence, in addition to apoptosis. Western blot analysis confirmed reduced expression of IRF1 in DBTRG cells following IRF1 knockdown, and Lamin B1 expression was also reduced (Figure 2C–E). Attenuated cell proliferation is a hallmark of cellular senescence [11,39]. As expected, the proportion of PH3-positive DBTRG cells was significantly decreased after knockdown of IRF1 (Figure 3A–C). The growth rate of DBTRG cells was also reduced following IRF1 knockdown, compared with control cells (Supplementary Figure S4A). Moreover, ELISA results revealed that the level of MMP-3 (a SASP factor) in the supernatant was elevated after IRF1 knockdown (Figure 3D), suggesting enhanced secretion of SASP factors. Altogether, these results indicate that downregulation of IRF1 promotes senescence of GBM cells.

### 3.3. Overexpression of IRF1 Partially Restores TMZ-Induced Senescence of GBM Cells

**Figure 4:**
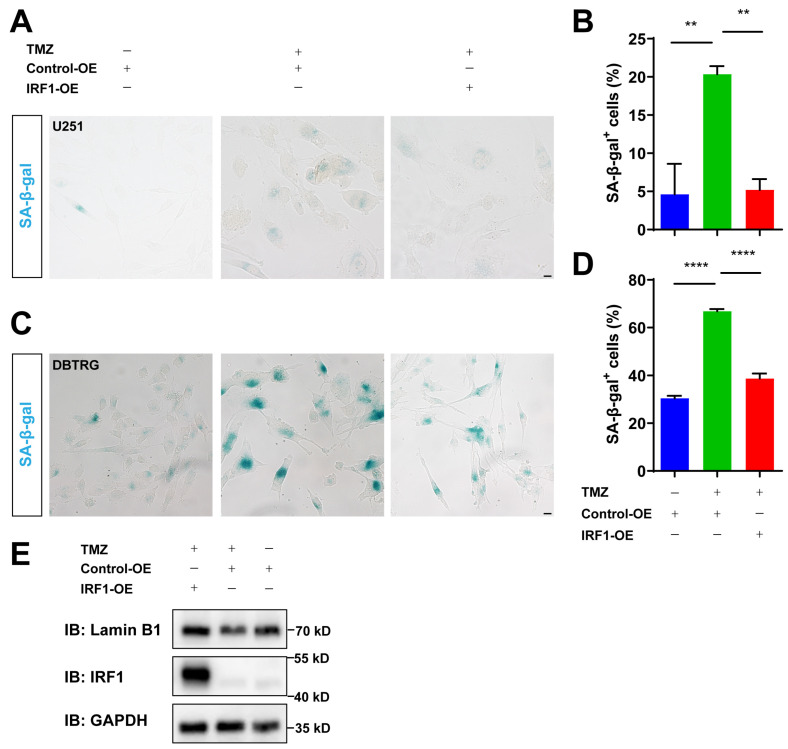
Overexpression of IRF1 reverses TMZ-induced senescence and SASP of GBM cells. (**A**) Representative SA-β-gal staining micrographs of U251 cells infected with control lentivirus (control-OE), TMZ-induced senescent U251 cells infected with control lentivirus (control-OE), and TMZ-induced senescent U251 cells infected with IRF1-overexpressing lentivirus (IRF1-OE) for 48–96 h. (**B**) Quantitative analysis of the proportion of SA-β-gal^+^ cells (*n* = 3). (**C**) Representative SA-β-gal staining micrographs of DBTRG cells infected with control lentivirus (control-OE), TMZ-induced senescent DBTRG cells infected with control lentivirus (control-OE), and TMZ-induced senescent DBTRG cells infected with IRF1-overexpressing lentivirus (IRF1-OE) for 48–96 h. (**D**) Quantitative analysis of the proportion of SA-β-gal^+^ cells (*n* = 4). (**E**) Expression of IRF1 and Lamin B1 in DBTRG cells infected with control lentivirus (control-OE), TMZ-induced senescent DBTRG cells infected with control lentivirus (control-OE), and TMZ-induced senescent DBTRG cells infected with IRF1-overexpressing lentivirus (IRF1-OE) for 48–96 h, as detected by Western blot. One-way ANOVA. ** *p* < 0.01, **** *p* < 0.0001. Scale bar, 20 μm.

We next investigated whether upregulation of IRF1 could inhibit senescence of GBM cells. As expected, overexpression of IRF1 by using the IRF1-overexpressing lentivirus partially rescued the TMZ-induced decrease in the proportion of SA-β-gal-positive U251 and DBTRG cells (Figure 4A–D). Furthermore, overexpression of IRF1 indeed partially reversed the decrease in Lamin B1 expression in TMZ-induced senescent DBTRG cells (Figure 4E). These results showed that overexpression of IRF1 partially restores TMZ-triggered senescence in GBM cells.

### 3.4. IRF1 Regulates the Expression of OAS2

**Figure 5:**
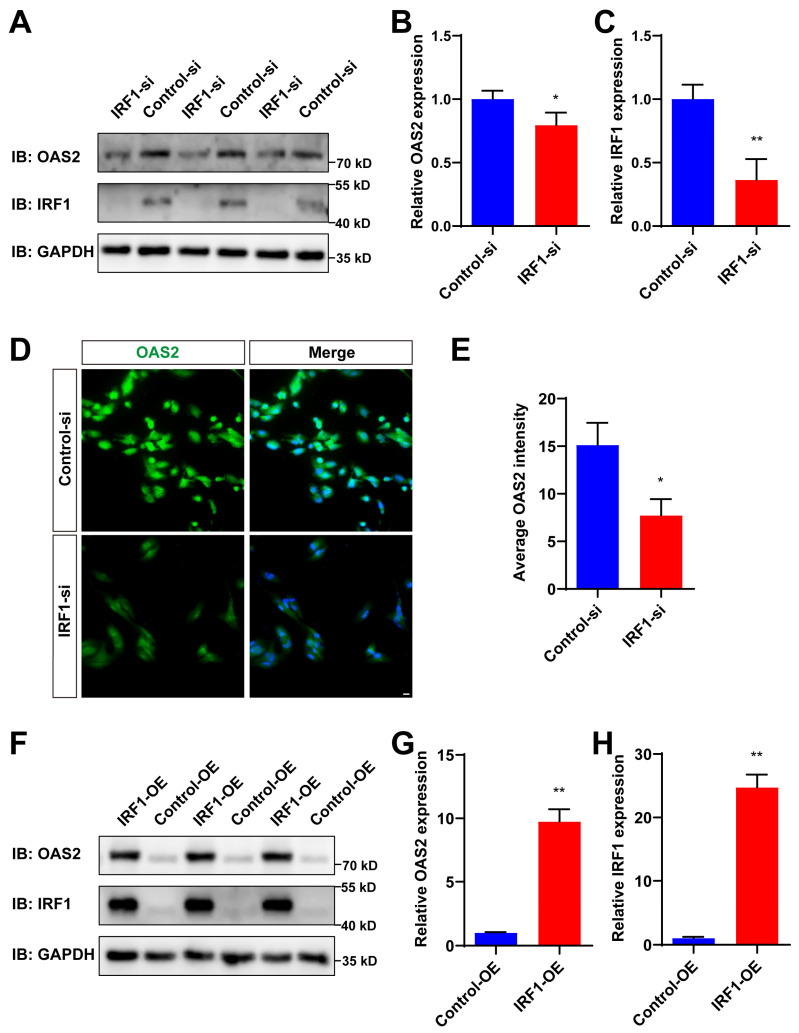
IRF1 regulates the expression of 2′,5′-oligoadenylate synthetase 2 (OAS2). (**A**) Expression of OAS2 in DBTRG cells transfected with either control-siRNA or IRF1-siRNA for 48–96 h, as detected by Western blot. (**B**,**C**) Quantitative analysis of IRF1 and OAS2 expression (*n* = 4). (**D**) Representative OAS2 (green) immunostaining images of DBTRG cells transfected with either control-siRNA or IRF1-siRNA for 48–96 h. (**E**) Quantitative analysis of average fluorescence intensity of OAS2 (*n* = 6). (**F**) Expression of IRF1 and OAS2 in DBTRG cells infected with either control lentivirus (control-OE) or IRF1-overexpressing lentivirus (IRF1-OE) for 48–96 h, as detected by Western blot. (**G**,**H**) Quantitative analysis of IRF1 and OAS2 expression (*n* = 4). Nuclei were counterstained with DAPI (blue). Student’s *t*-test. * *p* < 0.05, ** *p* < 0.01. Scale bar, 20 μm.

Previous studies have suggested that OAS2, which functions crucially in innate immunity [29,40,41], is one of the downstream targets of IRF1 [25,26,27]. We further tested whether IRF1 downregulation in senescent GBM cells leads to changes in OAS2 expression. The decrease in OAS2 protein level after IRF1 knockdown was confirmed by Western blot (Figure 5A–C). Immunostaining also showed reduced expression of OAS2 in DBTRG cells after IRF1 knockdown (Figure 5D,E). In contrast, Western blot results revealed an increase in OAS2 expression following IRF1 overexpression (Figure 5F–H). These data indicate that IRF1 modulates the expression of OAS2.

### 3.5. OAS2 Is Downregulated in TMZ-Induced Senescent GBM Cells

**Figure 6:**
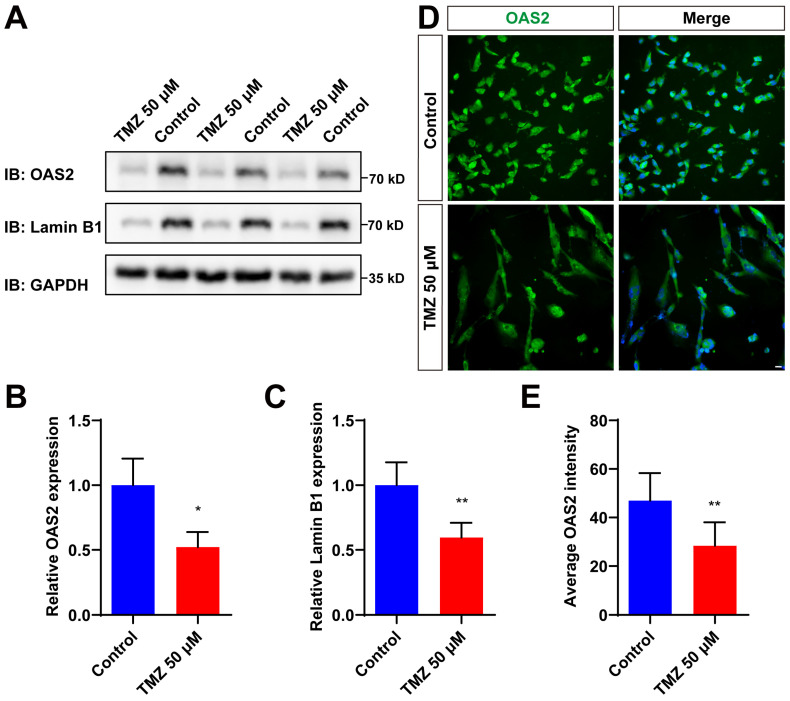
OAS2 is downregulated in TMZ-induced senescent GBM cells. (**A**) Expression of OAS2 and Lamin B1 in DBTRG cells exposed to either DMSO or 50 μM TMZ over 4–8 days, as detected by Western blot. (**B**,**C**) Quantitative analysis of OAS2 and Lamin B1 expression (*n* = 8). (**D**) Representative OAS2 (green) immunostaining images of DBTRG cells exposed to either DMSO or 50 μM TMZ over 4–8 days. (**E**) Quantitative analysis of average fluorescence intensity of OAS2 (*n* = 5). Nuclei were counterstained with DAPI (blue). Student’s *t*-test. * *p* < 0.05, ** *p* < 0.01. Scale bar, 20 μm.

To confirm the function of OAS2 in TMZ-induced senescent GBM cells, we next detected the expression of OAS2 after senescence induction. Western blot results exhibited decreased expression of OAS2 and Lamin B1 in 50 μM TMZ-induced senescent DBTRG cells (Figure 6A–C and Supplementary Figure S5A). Immunofluorescent staining experiment further validated the reduction in OAS2 expression in these senescent DBTRG cells (Figure 6D,E). This evidence indicates that OAS2 expression is decreased in TMZ-triggered senescent GBM cells.

### 3.6. Knockdown of OAS2 Induces Senescence and Promotes the Secretion of SASP Factors of GBM Cells

**Figure 7:**
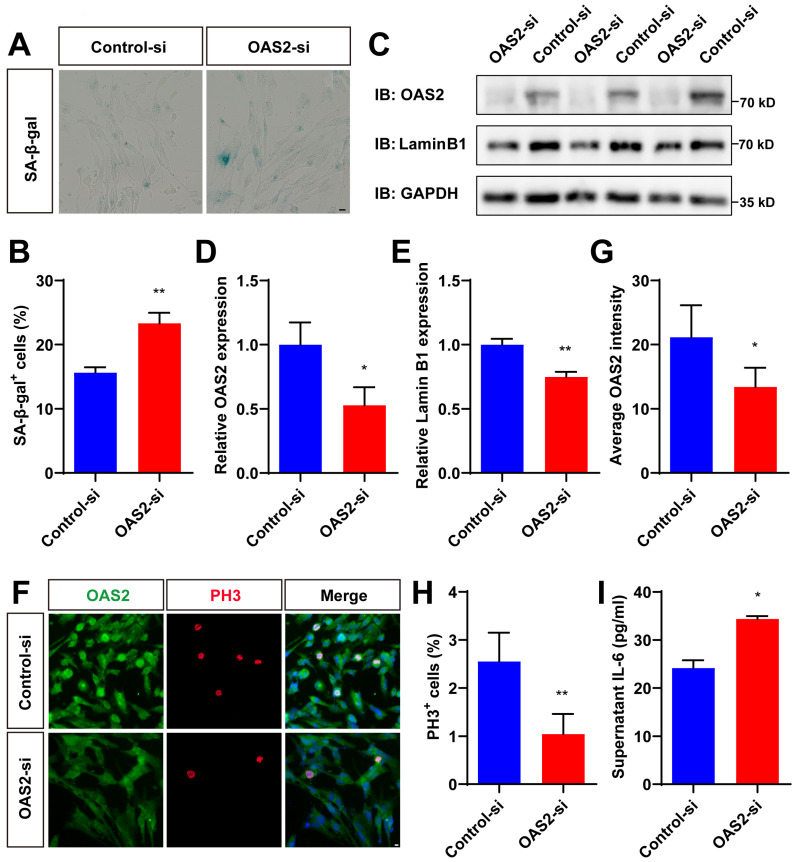
Down-regulation of OAS2 promotes senescence of GBM cells. (**A**) Representative SA-β-gal staining micrographs of DBTRG cells transfected with either control-siRNA or OAS2-siRNA for 48–96 h. (**B**) Quantitative analysis of the proportion of SA-β-gal^+^ DBTRG cells (*n* = 4). (**C**) Expression of OAS2 and Lamin B1 in DBTRG cells transfected with control-siRNA or OAS2-siRNA for 48–96 h, as detected by Western blot. (**D**,**E**) Quantitative analysis of Lamin B1 and OAS2 expression (*n* = 5). (**F**) Representative OAS2 (green) and PH3 (red) immunostaining images of DBTRG cells transfected with either control-siRNA or OAS2-siRNA for 48–96 h. (**G**,**H**) Quantitative analysis of average fluorescence intensity of OAS2 (*n* = 7) and the proportion of PH3^+^ cells (*n* = 4). (**I**) Measurement of IL-6 level in the supernatant of DBTRG cells transfected with either control-siRNA or OAS2-siRNA for 48–96 h, as detected by ELISA (*n* = 7). Nuclei were counterstained with DAPI (blue). Student’s *t*-test. * *p* < 0.05, ** *p* < 0.01. Scale bar, 20 μm.

To confirm the function of OAS2 in GBM senescence, OAS2-specific siRNA was synthesized to knock down OAS2 [29]. As shown in Figure 7A,B, an increase in the proportion of SA-β-gal-positive DBTRG cells was detected after OAS2 knockdown, compared with control cells, indicating that OAS2 knockdown promoted cellular senescence. Decreased expression of OAS2 in DBTRG cells after OAS2 knockdown was shown by Western blot, and Lamin B1 expression was also reduced in these OAS2-knockdown DBTRG cells (Figure 7C–E). Moreover, the proportion of PH3-positive DBTRG cells was significantly decreased after knockdown of OAS2 (Figure 7F–H). The growth rate of DBTRG cells was also reduced following knockdown of OAS2, compared with control cells (Supplementary Figure S6A). In addition, ELISA results showed that the level of IL-6 (a SASP factor) in the supernatant was elevated after OAS2 knockdown (Figure 7I). Collectively, this evidence suggests that the downregulation of OAS2 promotes senescence of GBM cells.

## 4. Discussion

**Figure 8:**
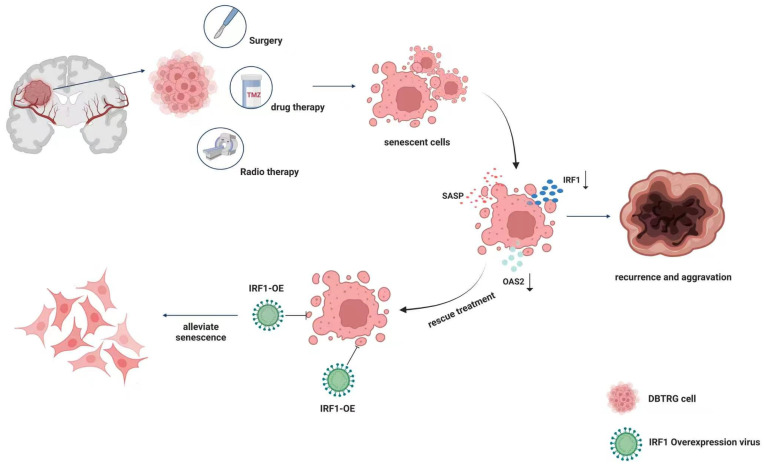
A working model illustrating the function of IRF1 in TMZ-induced senescent GBM cells. The expression of IRF1 in TMZ-treated GBM cell lines decreases, which then downregulates OAS2, leading to the secretion of SASP components and ultimately promoting senescence of GBM cells. Overexpression of IRF1 alleviates the senescence of TMZ-treated GBM cells.

In this work, we provide evidence supporting a role for IRF1 in GBM senescence and propose a mechanistic framework (Figure 8). Specifically, IRF1 expression decreases in TMZ-treated GBM cells, which leads to downregulation of OAS2, subsequent secretion of SASP components, and ultimately promotes senescence of GBM cells. Overexpression of IRF1 alleviates the senescence of TMZ-treated GBM cells. Thus, a new mechanistic pathway by which IRF1 regulates senescence of GBM cells was identified in this study, providing a new target and direction for therapeutic intervention against GBM.

Several cutting-edge studies have investigated the complex role of IRF1 in cellular senescence [21,22,23,24]. For example, after irradiation, the expression of IRF1 is increased in human GBM cells and human structural cells [21,22]. Conversely, knockdown of IRF1 rescues radiation-induced senescence in skin cells [21]. IRF1 expression is also increased in human melanoma cells after interferon treatment, and interferon treatment induces cellular senescence of endothelial cells. These findings suggest that IRF1 promotes cellular senescence. However, Cho et al. reported that knockdown of IRF1 increases senescence of chondrocytes treated with bleomycin by blocking DNA-damage repair, whereas overexpression of IRF1 rescues bleomycin-induced senescence of chondrocytes [24]. In agreement with this, we found that IRF1 inhibits senescence of GBM cells, as shown by enhanced SA-β-gal activity, increased SASP secretion, decreased expression of Lamin B1, and attenuated proliferation upon IRF1 knockdown. Variations in species and cell types could account for the conflicting roles of IRF1 during cellular senescence. For example, upregulation of IRF1 protein occurs in skin tissues of rodents and monkeys at day three and day seven post-irradiation, but downregulation is detected in skin tissues of human patients after fractional radiotherapy [21]. The same study also showed that the expression of IRF1 varies across different cell types in patients after irradiation; for example, the mRNA level of IRF1 increases in mast cells, decreases in fibroblasts, and remains unchanged in some other cell types [21].

Furthermore, IRF1 expression may change dynamically over time, possibly depending on the detection time point. It has been reported that a single high dose of radiation causes a decrease in the mRNA level of IRF1 at one-hour post-irradiation; however, the level peaks at 2–4 h post-irradiation and then declines again at 12 h post-irradiation [21]. In our study, we only examined the expression of IRF1 at 4–8 days after TMZ treatment and ignored earlier or later time points. It is possible that the expression of IRF1 exhibits different changes at other time points.

Additionally, the changes and actions of IRF1 may be context-dependent. For example, fractional radiation and a single high dose of radiation induce different alterations in activity and mRNA level of IRF1 [21]. After all, for GBM cells, irradiation and TMZ represent different stimuli and can activate distinct signaling pathways [7,9,10,15,42].

It has been reported that knockout of IRF1 increases the production of certain SASP components, such as MMP-13 and IL-6 [24]. In our study, we observed that knockdown of IRF1 increases the secretion of MMP-3, which is consistent with these reports. However, the SASP secretome is also context-dependent [43]. One study revealed that knockout of IRF1 decreases the mRNA level of IL-1, while overexpression of IRF1 increases the mRNA level of IL-1 [21]. Therefore, the specific SASP secretome of TMZ-induced senescent cells requires further detailed investigation.

Moreover, suppression of the PI3K-AKT signaling has been shown to promote senescence of GBM cells [44], while its activation inhibits the degradation of IRF1 [45], further suggesting that a high level of IRF1 may inhibit the senescence of GBM cells.

We also noticed that IRF1 was highly expressed in GBM patients in the CGGA database, compared to patients with low-grade glioma, despite IRF1 being considered a tumor suppressor. On the one hand, elevated IRF1 expression may inhibit senescence and potentially promote rapid proliferation of GBM cells, thereby aggravating GBM. On the other hand, as mentioned above, knockdown of IRF1 was found to induce apoptosis in a subset of GBM cells in our study. From this perspective, a high level of IRF1 in recurrent GBM patients may inhibit apoptosis, thereby also contributing to GBM progression. Nevertheless, the role of IRF1 in GBM development is complex and warrants further investigation.

Transcriptome analysis, qRT-PCR and luciferase assays showed that OAS2 is one of the downstream targets of IRF1 [25,26,27]. As an interferon-stimulated gene, *oas2* encodes a key enzyme and plays a central role in innate immune recognition [28,29]. Integrated multi-omics analyses have shown a correlation between OAS2 expression and cellular senescence [30]; in ultraviolet radiation-induced senescent human primary melanocytes, the expression of OAS2 increases significantly [31]. These findings appear to conflict with our results. Different cell types may account for this inconsistency. Nevertheless, the changes in signaling pathway are complicated and context-dependent, and the role of OAS2 in cellular senescence requires further investigation.

In conclusion, our work revealed that IRF1 prevents senescence of GBM cells by regulating OAS2, thereby identifying a new role and mechanism of IRF1 in TMZ-induced senescence of GBM cells, and providing new perspectives for the therapeutic intervention of GBM.

## Data Availability

Data can be obtained by request from the corresponding author.

## Supplementary Materials

The following supporting information can be downloaded at: https://www.mdpi.com/article/10.3390/cells15131149/s1, Figure S1: TMZ induces senescence of GBM cells; Figure S2: TMZ decreases growth and proliferation of GBM cells; Figure S3: Expression of IRF1 following treatment with various concentrations of TMZ; Figure S4: IRF1 knockdown decreases the growth and induces hypertrophy of GBM cells; Figure S5: Expression of OAS2 following treatment with various concentrations of TMZ; Figure S6: OAS2 knockdown decreases the growth and induces hypertrophy of GBM cells.

## Abbreviations

The following abbreviations are used in this manuscript:

GBM	Glioblastoma
TIS	Therapy-induced senescence
TMZ	Temozolomide
IRF1	Interferon regulatory factor-1
SA-β-gal	Senescence-associated β-galactosidase
SASP	Senescence-associated secretory phenotype
OAS2	2′-5′-oligoadenylate synthetase 2
CNS	Central nervous system
MMP	Matrix metalloproteinases
siRNA	small interfering RNA
ELISA	Enzyme-linked immunosorbent assay
qRT-PCR	Quantitative real-time PCR
PH3	Phosphorylated histone H3
