# Supplementary material for: IRF1 Inhibits Therapy-Induced Senescence of Glioblastoma Cells Through OAS2
Source: Cells. 2026 Jun 24;15(13):1149. doi: 10.3390/cells15131149 (PMC13359778; doi:10.3390/cells15131149)
Supplement: Supplementary file 1 [file cells-15-01149-s001.zip › cells-4323898-supplementary.pdf]

**Supplementary materials**

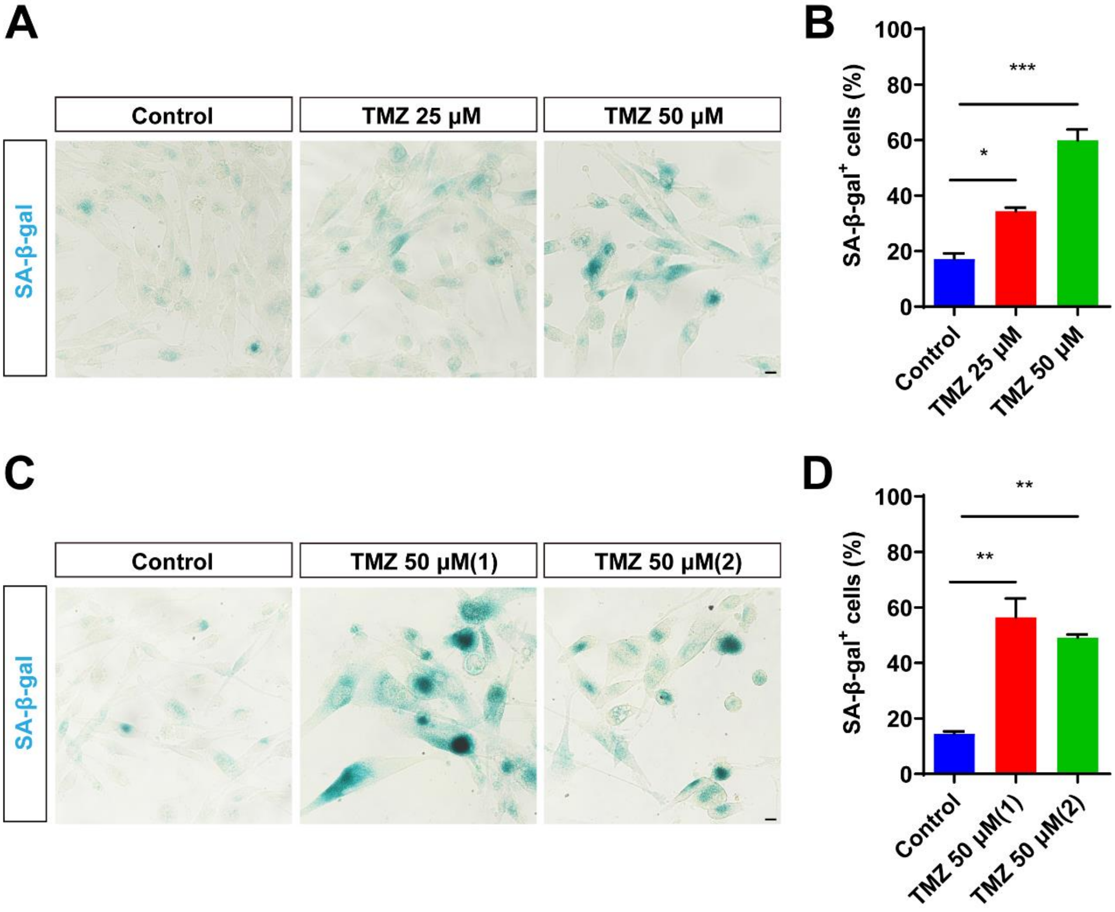


**Figure S1.** TMZ Induces Senescence of GBM Cells**. (A)** Representative SA-β-gal staining micrographs of DBTRG cells exposed to DMSO, 25 μM TMZ or 50 μM TMZ over 4–8 days. **(B)** Quantitative analysis of the proportion of SA-β-gal+ DBTRG cells ($n$ = 3). **(C)** Representative SA-β-gal staining micrographs of DBTRG cells exposed to DMSO (control), 50 μM TMZ once or 50 μM TMZ twice over a period of 4–8 days. **(D)** Quantitative analysis of the proportion of SA-β-gal+ DBTRG cells ($n$ = 3). One-way ANOVA. * $p < 0.05$, ** $p < 0.01$, *** $p < 0.001$. Scale bar, 20 μm.

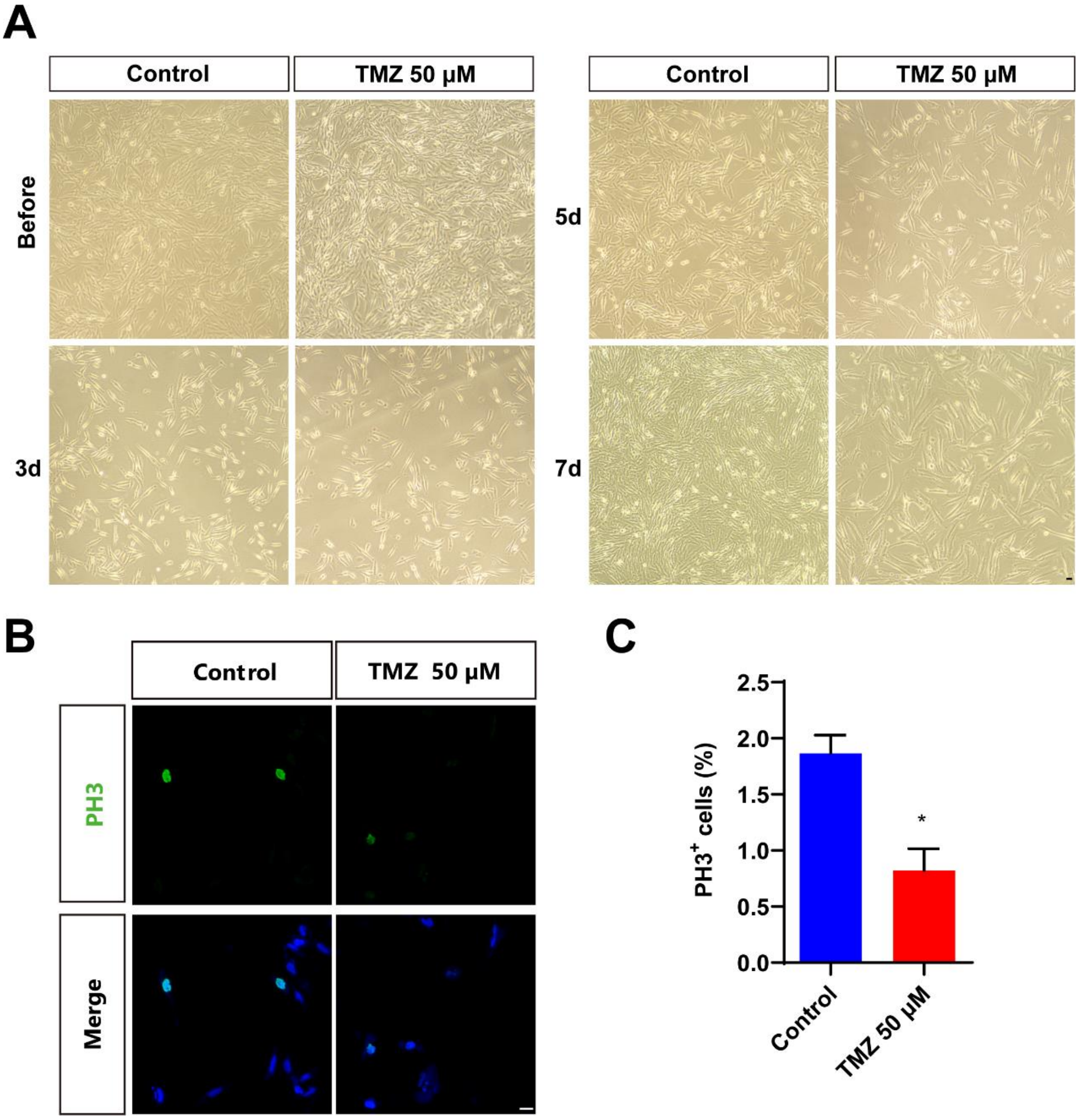


**Figure S2.** TMZ Decreases Growth and Proliferation of GBM Cells**. (A)** Bright-field micrographs of DBTRG cells before treatment and after treatment with either DMSO or 50 μM TMZ on day 3, day 5, and day 7. **(B)** Representative PH3 (green) staining micrographs of DBTRG cells exposed to either DMSO or 50 μM TMZ over 4–8 days. **(C)** Quantitative analysis of the proportion of PH3$^{+}$ DBTRG cells ($n = 3$). Nuclei were counterstained with DAPI (blue). Student's t-test. * $p < 0.05$. Scale bar, 20 μm.

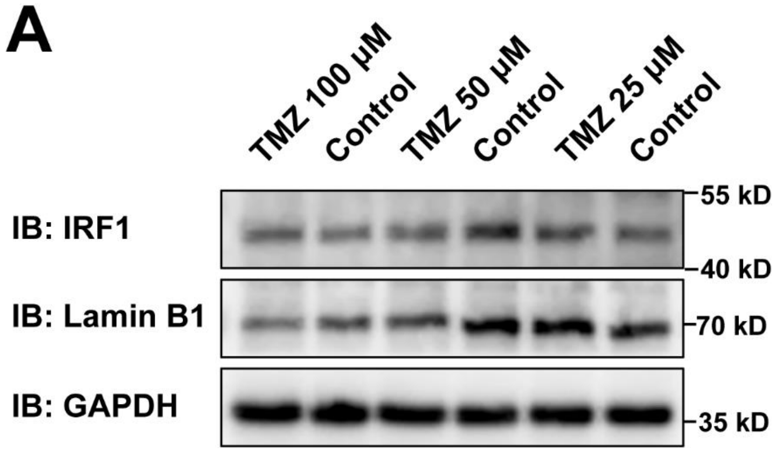


**Figure S3.** Expression of IRF1 Following Treatment with Various Concentrations of TMZ. **(A)** Expression of IRF1 and Lamin B1 in control and TMZ-treated groups at various concentrations for 4–8 days, as detected by Western blot.

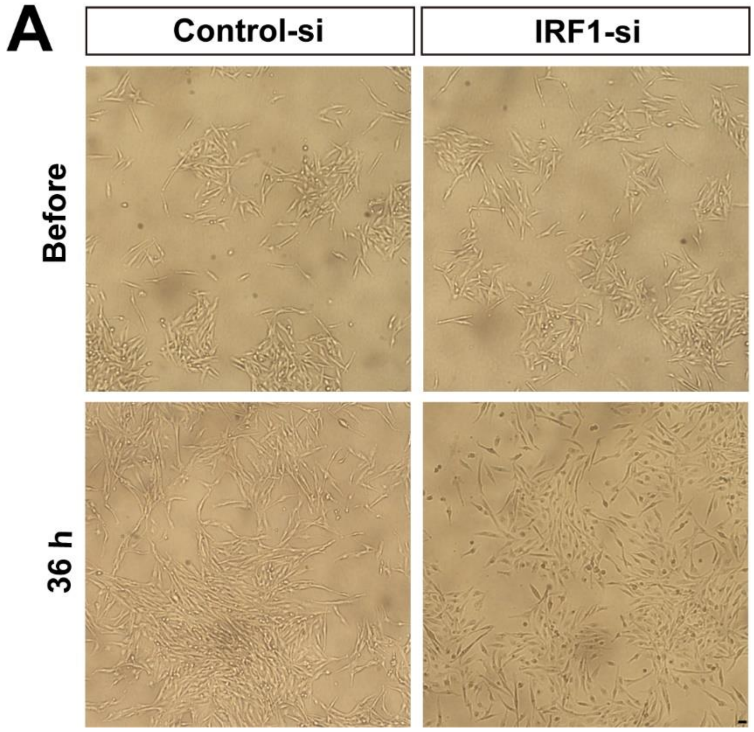


**Figure S4.** IRF1 Knockdown Decreases the Growth and Induces Hypertrophy of GBM Cells. **(A)** Bright-field micrographs of DBTRG cells before transfection and 36 h post-transfection with either control-siRNA or IRF1-siRNA. Scale bar, 20 μm.

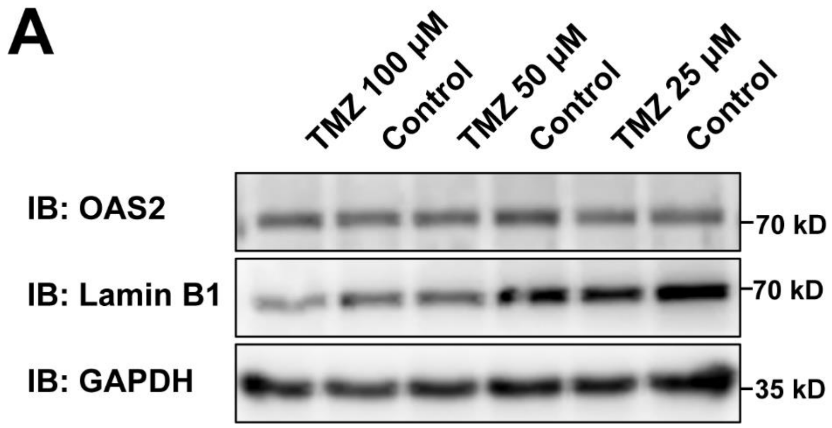


**Figure S5.** Expression of OAS2 Following Treatment with Various Concentrations of TMZ. **(A)** Expression of OAS2 and Lamin B1 in control and TMZ-treated groups at various concentrations for 4–8 days, as detected by Western blot.

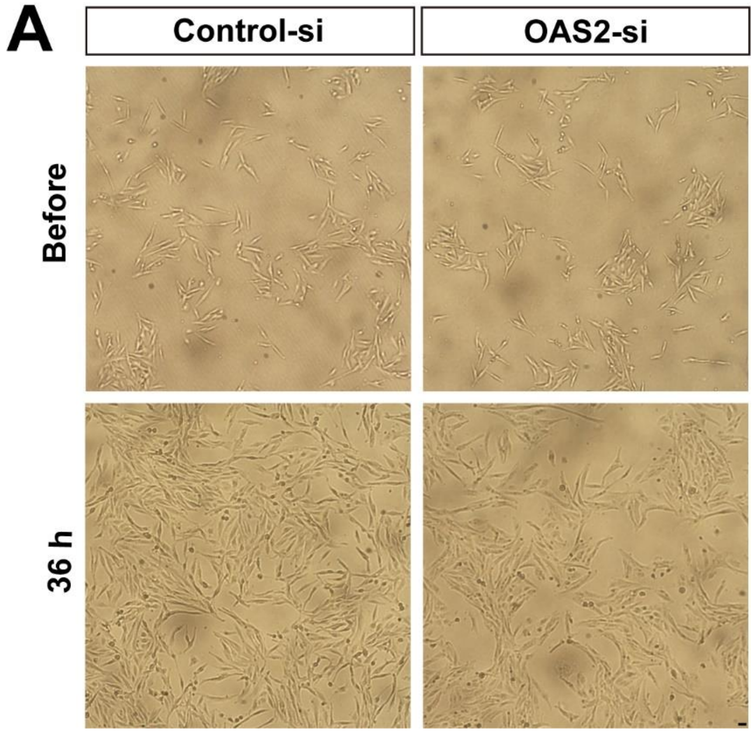


**Figure S6.** OAS2 Knockdown Decreases the Growth and Induces Hypertrophy of GBM Cells. **(A)** Bright-field micrographs of DBTRG cells before transfection and 36 h post- transfection with either control-siRNA or OAS2-siRNA. Scale bar, 20 μm.
